# Supplementary material for: Decoding of columnar-level organization across cortical depth using BOLD- and CBV-fMRI at 7 T
Source: bioRxiv. 2025 Aug 27:2023.09.28.560016. Preprint. [Version 2] doi: 10.1101/2023.09.28.560016 (PMC12407697; doi:10.1101/2023.09.28.560016)
Supplement: Supplement 1 [file NIHPP2023.09.28.560016v2-supplement-1.pdf]

bioRxiv preprint doi: https://doi.org/10.1101/2023.09.28.560016; this version posted August 27, 2025. The copyright holder for this preprint (which was not certified by peer review) is the author/funder, who has granted bioRxiv a license to display the preprint in perpetuity. It is made available under aCC-BY 4.0 International license.

Supplementary Information

Decoding of columnar-level organization across cortical depth using BOLD- and CBV-fMRI at 7 T

Daniel Haenelt [1,2] ✉, **Denis Chaimow** [1], **Marianna Elisa Schmidt** [1,3], **Shahin Nasr** [4,5], **Nikolaus Weiskopf** [1,6,7], **Robert Trampel** [1]

[1]Department of Neurophysics, Max Planck Institute for Human Cognitive and Brain Sciences, 04103 Leipzig, Germany; [2]International Max Planck Research School on Neuroscience of Communication: Function, Structure, and Plasticity, 04103 Leipzig, Germany; [3]Max Planck School of Cognition, 04103 Leipzig, Germany; [4]Athinoula A. Martinos Center for Biomedical Imaging, Massachusetts General Hospital, Charlestown, MA 02129, USA; [5]Department of Radiology, Harvard Medical School, Boston, MA 02114, USA; [6]Felix Bloch Institute for Solid State Physics, Faculty of Physics and Earth Sciences, Leipzig University, 04103 Leipzig, Germany; [7]Wellcome Centre for Human Neuroimaging, Institute of Neurology, University College London, London WC1N 3AR, UK

bioRxiv preprint doi: https://doi.org/10.1101/2023.09.28.560016; this version posted August 27, 2025. The copyright holder for this preprint (which was not certified by peer review) is the author/funder, who has granted bioRxiv a license to display the preprint in perpetuity. It is made available under aCC-BY 4.0 International license.

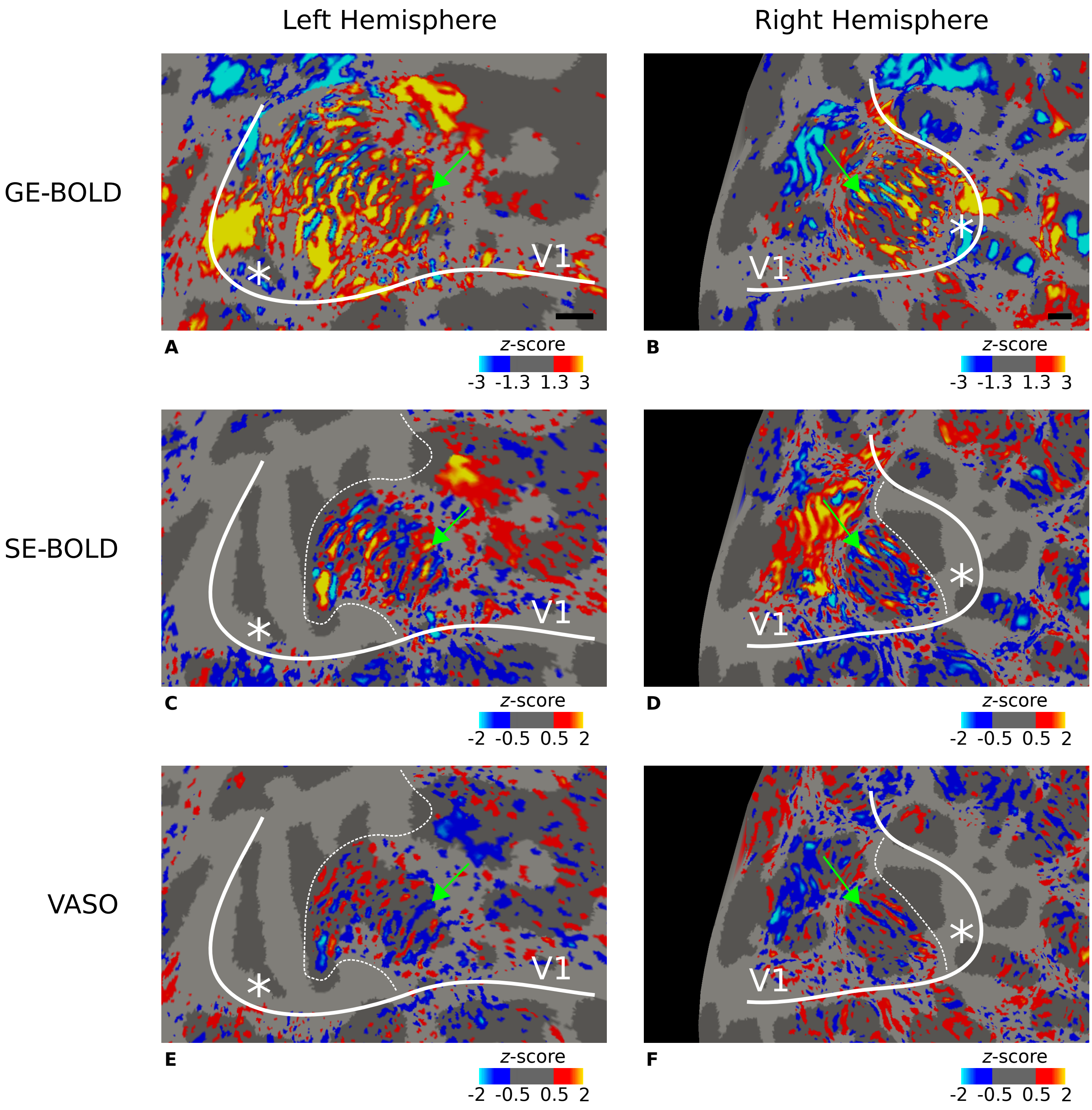

Supplementary Figure 1. Ocular dominance columns (ODCs) from subject 1. Thresholded activation maps (contrast left eye > right eye) are shown for the left and right hemisphere, respectively, for GE-BOLD **(A–B)**, SE-BOLD **(C–D)**, and VASO **(E–F)**. Data were averaged across sessions, sampled at mid-cortical depth, and shown on flattened surfaces. Similarities between maps are evident. Green arrows point to columns that were reproducibly activated between scanning sessions. This participant was left eye dominant. Note that VASO has an inverted contrast compared to BOLD. Black lines in **A** and **B** show scale bars (5 mm), respectively—other details as in ***Figure 2***.

bioRxiv preprint doi: https://doi.org/10.1101/2023.09.28.560016; this version posted August 27, 2025. The copyright holder for this preprint (which was not certified by peer review) is the author/funder, who has granted bioRxiv a license to display the preprint in perpetuity. It is made available under aCC-BY 4.0 International license.

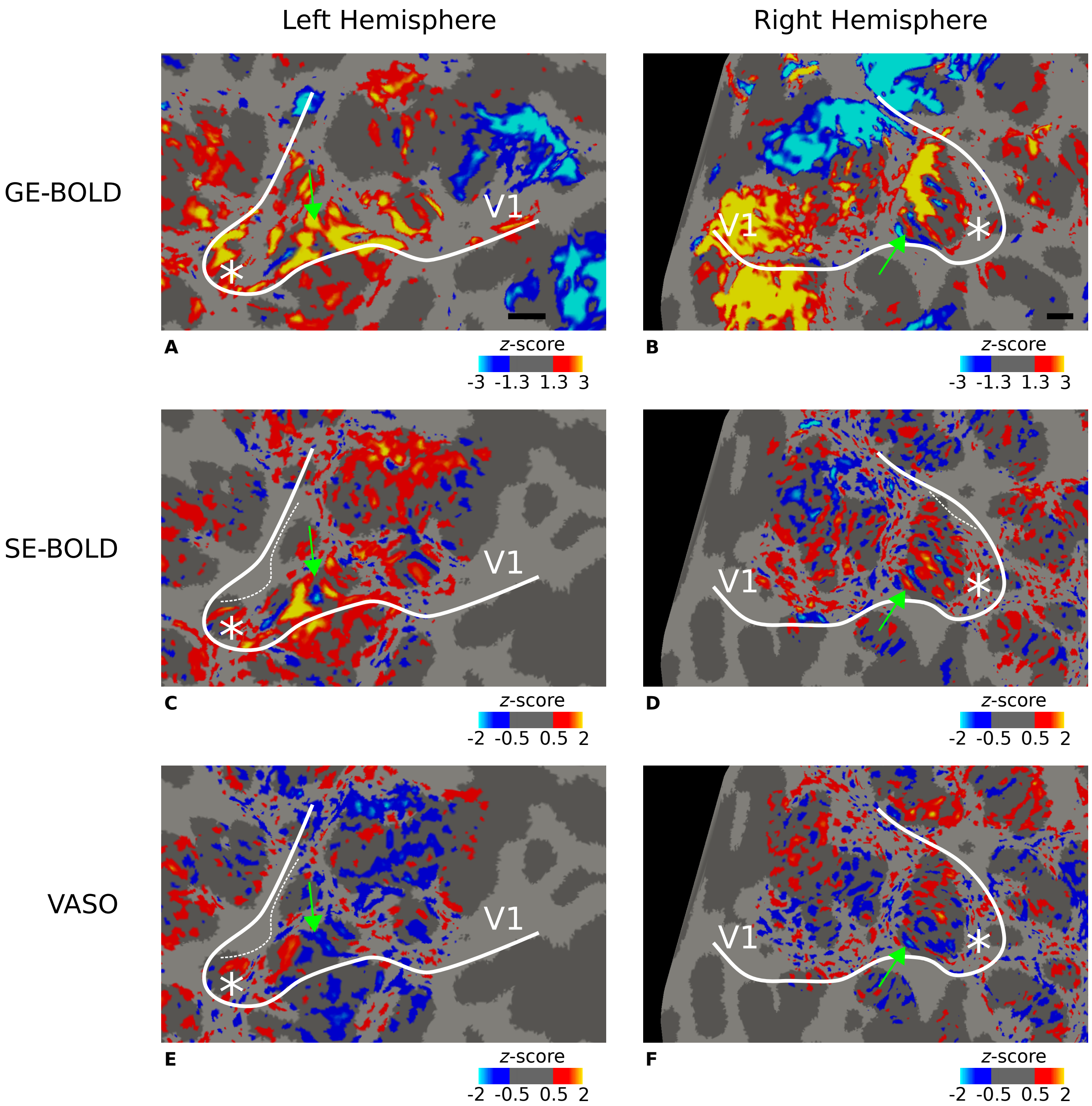

Supplementary Figure 2. Ocular dominance columns (ODCs) from subject 2. Thresholded activation maps (contrast left eye > right eye) are shown for the left and right hemisphere, respectively, for GE-BOLD **(A–B)**, SE-BOLD **(C–D)**, and VASO **(E–F)**. Data were averaged across sessions, sampled at mid-cortical depth, and shown on flattened surfaces. Similarities between maps are evident. Green arrows point to columns that were reproducibly activated between scanning sessions. This participant was right eye dominant. Note that VASO has an inverted contrast compared to BOLD. Black lines in **A** and **B** show scale bars (5 mm), respectively—other details as in ***Figure 2***.

bioRxiv preprint doi: https://doi.org/10.1101/2023.09.28.560016; this version posted August 27, 2025. The copyright holder for this preprint (which was not certified by peer review) is the author/funder, who has granted bioRxiv a license to display the preprint in perpetuity. It is made available under aCC-BY 4.0 International license.

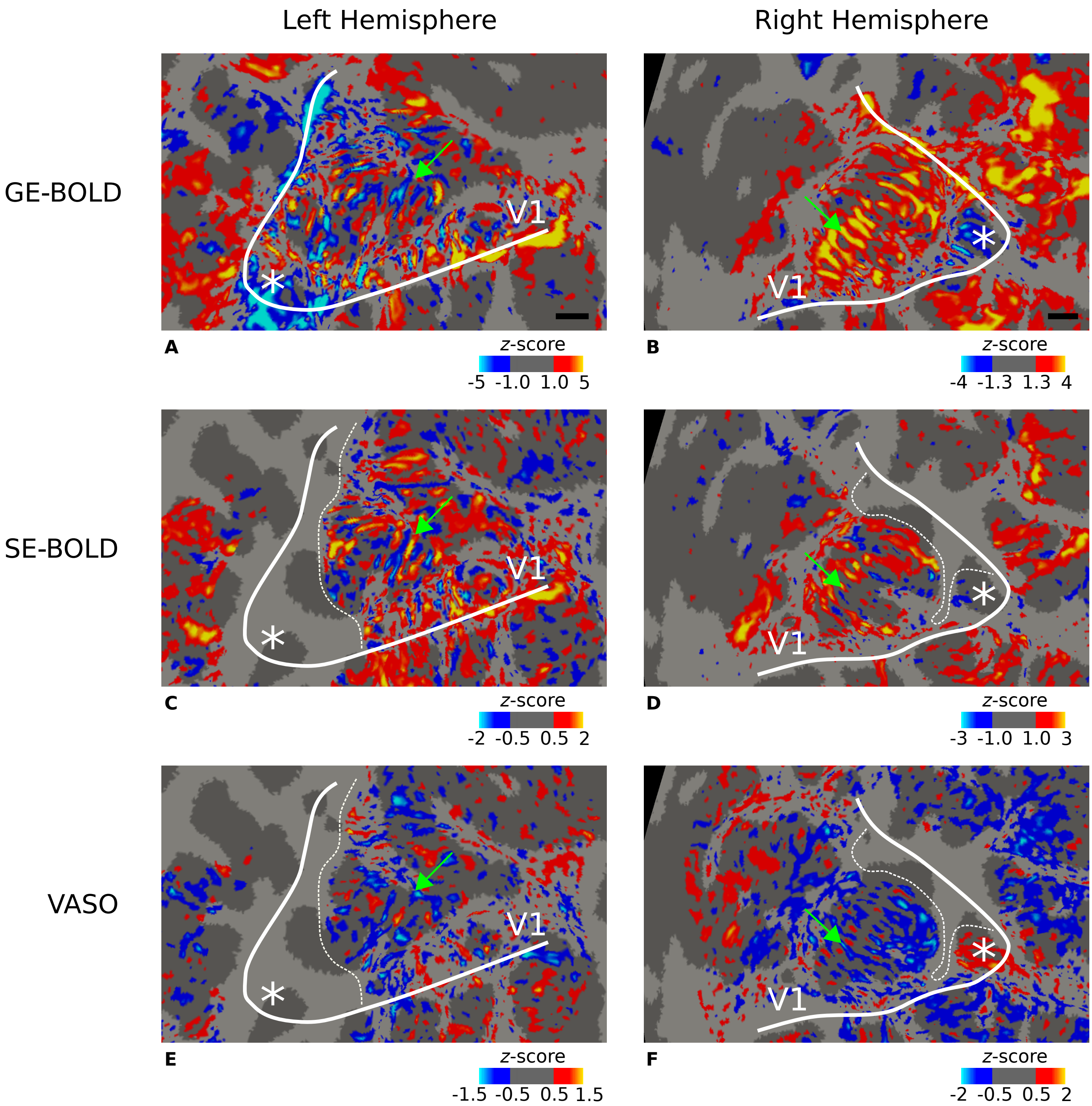

Supplementary Figure 3. Ocular dominance columns (ODCs) from subject 3. Thresholded activation maps (contrast left eye > right eye) are shown for the left and right hemisphere, respectively, for GE-BOLD **(A–B)**, SE-BOLD **(C–D)**, and VASO **(E–F)**. Data were averaged across sessions, sampled at mid-cortical depth, and shown on flattened surfaces. Similarities between maps are evident. Green arrows point to columns that were reproducibly activated between scanning sessions. This participant was left eye dominant. Note that VASO has an inverted contrast compared to BOLD. Black lines in **A** and **B** show scale bars (5 mm), respectively—other details as in ***Figure 2***.

bioRxiv preprint doi: https://doi.org/10.1101/2023.09.28.560016; this version posted August 27, 2025. The copyright holder for this preprint (which was not certified by peer review) is the author/funder, who has granted bioRxiv a license to display the preprint in perpetuity. It is made available under aCC-BY 4.0 International license.

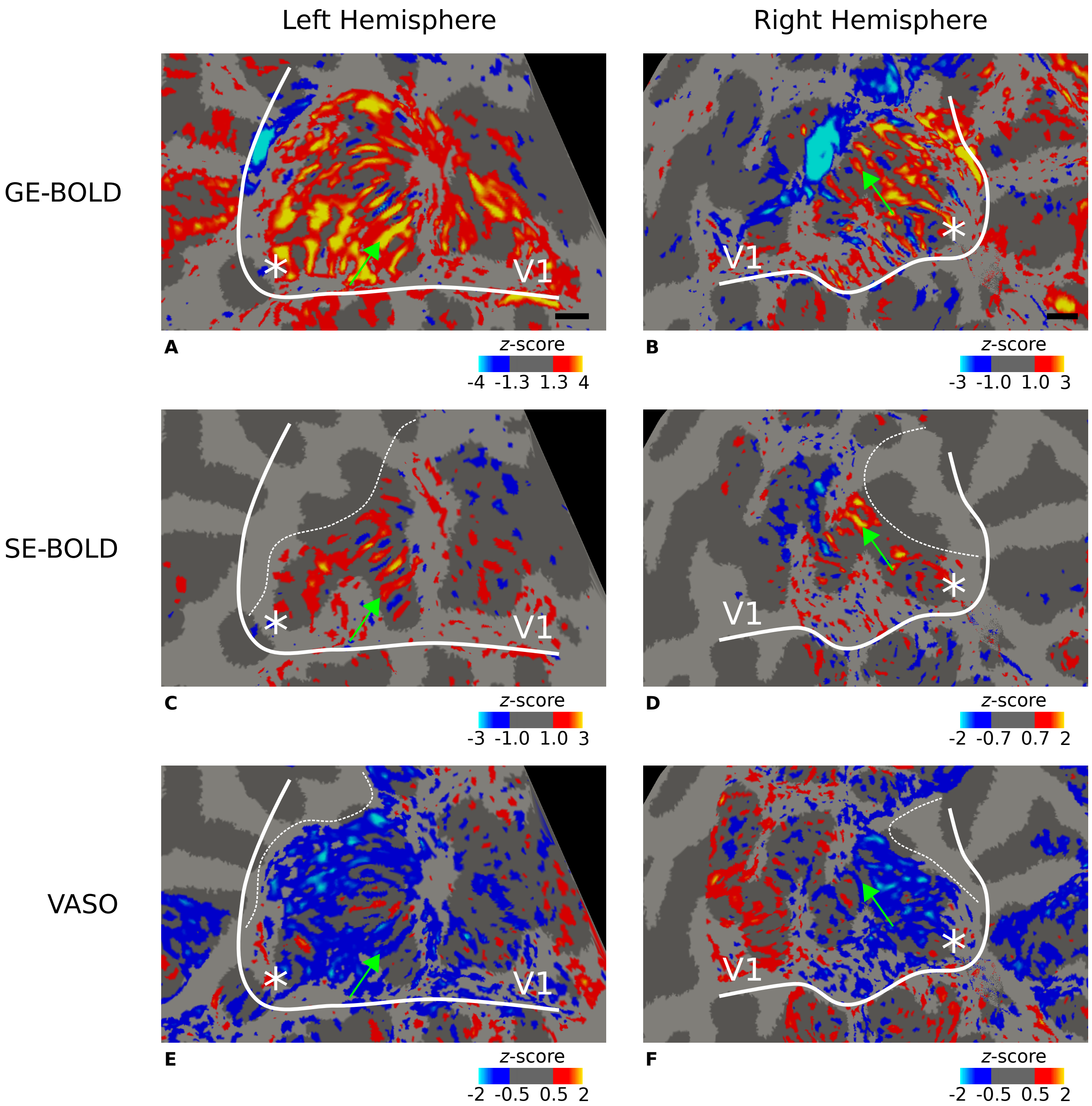

Supplementary Figure 4. Ocular dominance columns (ODCs) from subject 4. Thresholded activation maps (contrast left eye > right eye) are shown for the left and right hemisphere, respectively, for GE-BOLD **(A–B)**, SE-BOLD **(C–D)**, and VASO **(E–F)**. Data were averaged across sessions, sampled at mid-cortical depth, and shown on flattened surfaces. Similarities between maps are evident. Green arrows point to columns that were reproducibly activated between scanning sessions. This participant was right eye dominant. Note that VASO has an inverted contrast compared to BOLD. Black lines in **A** and **B** show scale bars (5 mm), respectively—other details as in ***Figure 2***.

bioRxiv preprint doi: https://doi.org/10.1101/2023.09.28.560016; this version posted August 27, 2025. The copyright holder for this preprint (which was not certified by peer review) is the author/funder, who has granted bioRxiv a license to display the preprint in perpetuity. It is made available under aCC-BY 4.0 International license.

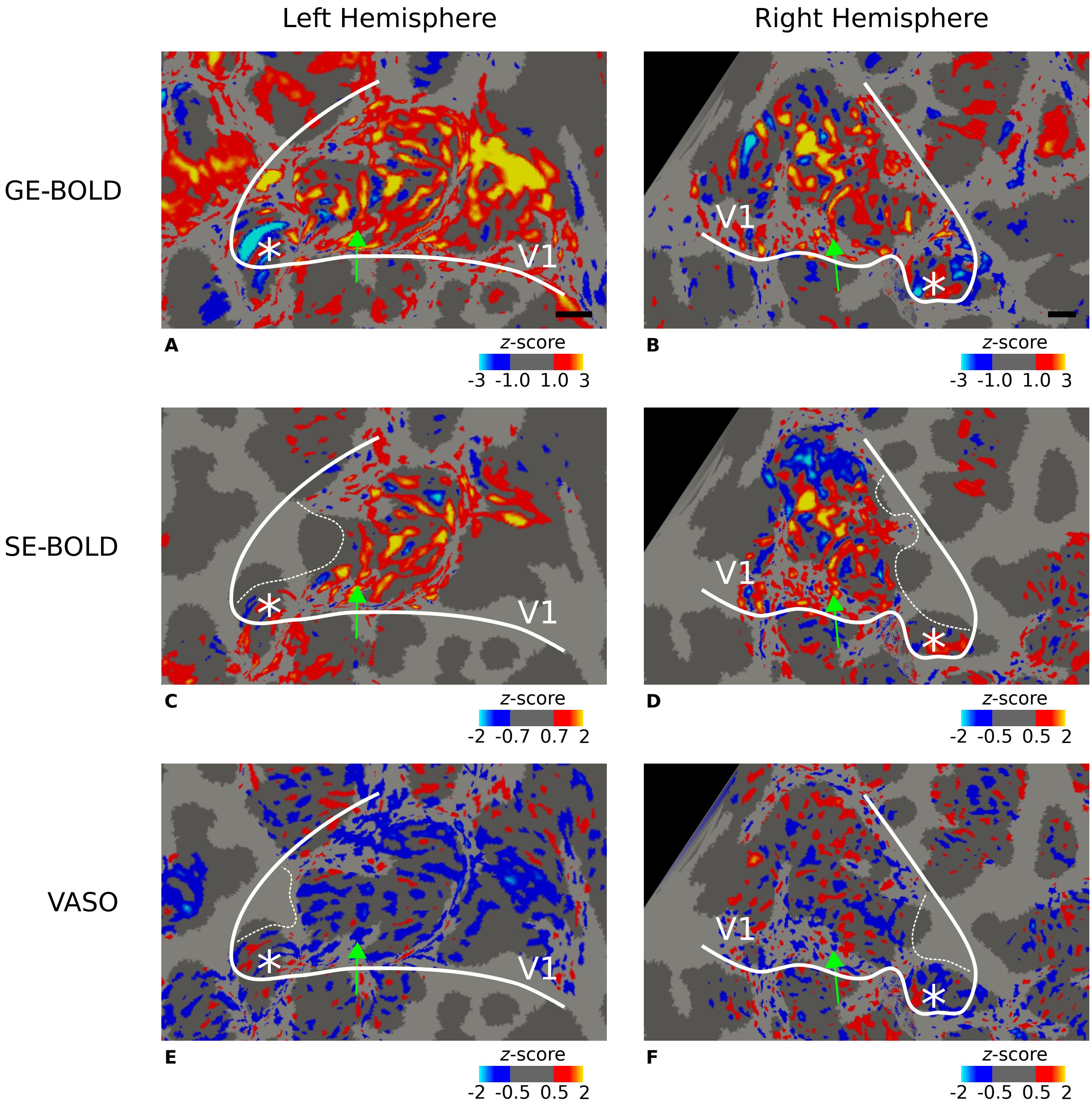

Supplementary Figure 5. Ocular dominance columns (ODCs) from subject 5. Thresholded activation maps (contrast left eye > right eye) are shown for the left and right hemisphere, respectively, for GE-BOLD **(A–B)**, SE-BOLD **(C–D)**, and VASO **(E–F)**. Data were averaged across sessions, sampled at mid-cortical depth, and shown on flattened surfaces. Similarities between maps are evident. Green arrows point to columns that were reproducibly activated between scanning sessions. This participant was left eye dominant. Note that VASO has an inverted contrast compared to BOLD. Black lines in **A** and **B** show scale bars (5 mm), respectively—other details as in ***Figure 2***.

bioRxiv preprint doi: https://doi.org/10.1101/2023.09.28.560016; this version posted August 27, 2025. The copyright holder for this preprint (which was not certified by peer review) is the author/funder, who has granted bioRxiv a license to display the preprint in perpetuity. It is made available under aCC-BY 4.0 International license.

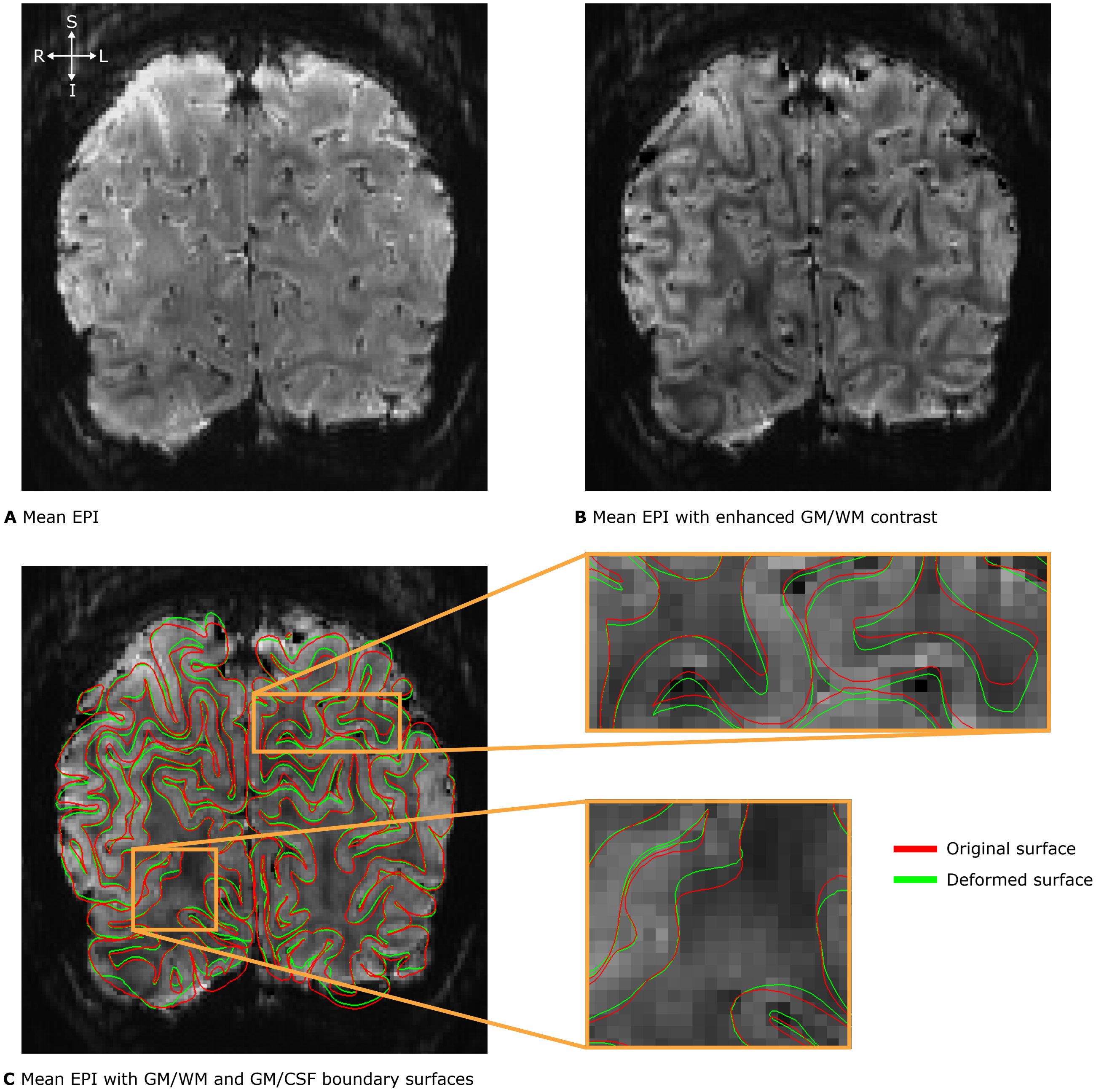

Supplementary Figure 6. Illustration of the GBB method. The method is used to enhance the alignment of cortical boundary surfaces based on an undistorted whole-brain anatomy to the cortical borders found in distorted functional images. **A** shows the temporal mean of the functional time series without task (GE-BOLD, 200 time points, subject 3) in coronal view that was acquired in the first session. **B** To enhance the GM/WM border and thereby increase the robustness of the proposed method, we weighted the temporal mean by its phase (see to Data preprocessing for detailed information) as usually done in susceptibility-weighted imaging methods. In **C**, the surfaces before (depicted in red) and after (depicted in green) alignment with the GBB method are presented. This technique is implemented in the GBB package (0.1.6, https://pypi.org/project/gbb/). The core idea of the method is to locally deform the GM/WM boundary surface iteratively until it reaches the GM/WM border found in the functional data. Each iteration starts by randomly selecting one vertex. Then, the vertex and its surrounding neighborhood is moved a small amount along the direction of increased GM/WM contrast scaled by a set step size. The change is evaluated by using the same cost function proposed in Greve and Fischl, 2009. Before alignment, surfaces are transformed into functional space via a rigid registration. From resulting vertex displacements of the GM/WM border, a deformation field is estimated that is then applied to the GM/CSF surface. The method improves spatial correspondence between the surfaces and the GM/WM boundaries observed in the functional images. GM: gray matter, WM: white matter, CSF: cerebrospinal fluid.

bioRxiv preprint doi: https://doi.org/10.1101/2023.09.28.560016; this version posted August 27, 2025. The copyright holder for this preprint (which was not certified by peer review) is the author/funder, who has granted bioRxiv a license to display the preprint in perpetuity. It is made available under aCC-BY 4.0 International license.

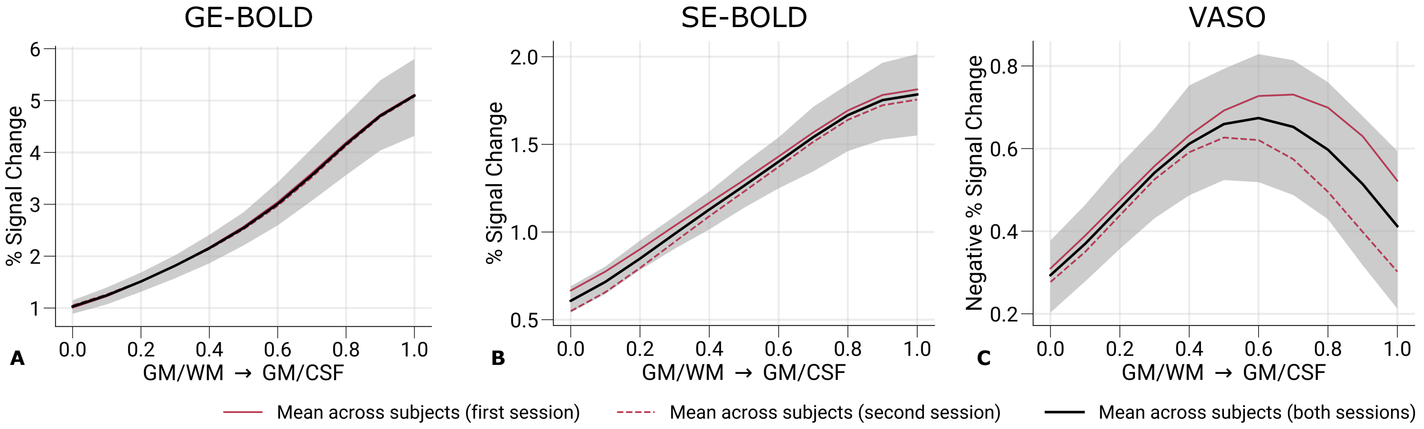

Supplementary Figure 7. Percent signal changes across cortical depth from whole V1. Mean percent signal changes (contrast: left eye and right eye > baseline) for GE-BOLD **(A)**, SE-BOLD **(B)**, and VASO **(C)** are shown across cortical depth. Contrary to ***Figure 5***, all V1 data inside the field of view across all scanning sessions were used. Compared to ***Figure 5***, lower percent signal changes and lower variability across participants can be identified. In **C**, the peak at mid-cortical depth is more pronounced. Note that we inverted the y-axis in **C** for consistency with **A** and **B**—other details as in ***Figure 5***.

bioRxiv preprint doi: https://doi.org/10.1101/2023.09.28.560016; this version posted August 27, 2025. The copyright holder for this preprint (which was not certified by peer review) is the author/funder, who has granted bioRxiv a license to display the preprint in perpetuity. It is made available under aCC-BY 4.0 International license.

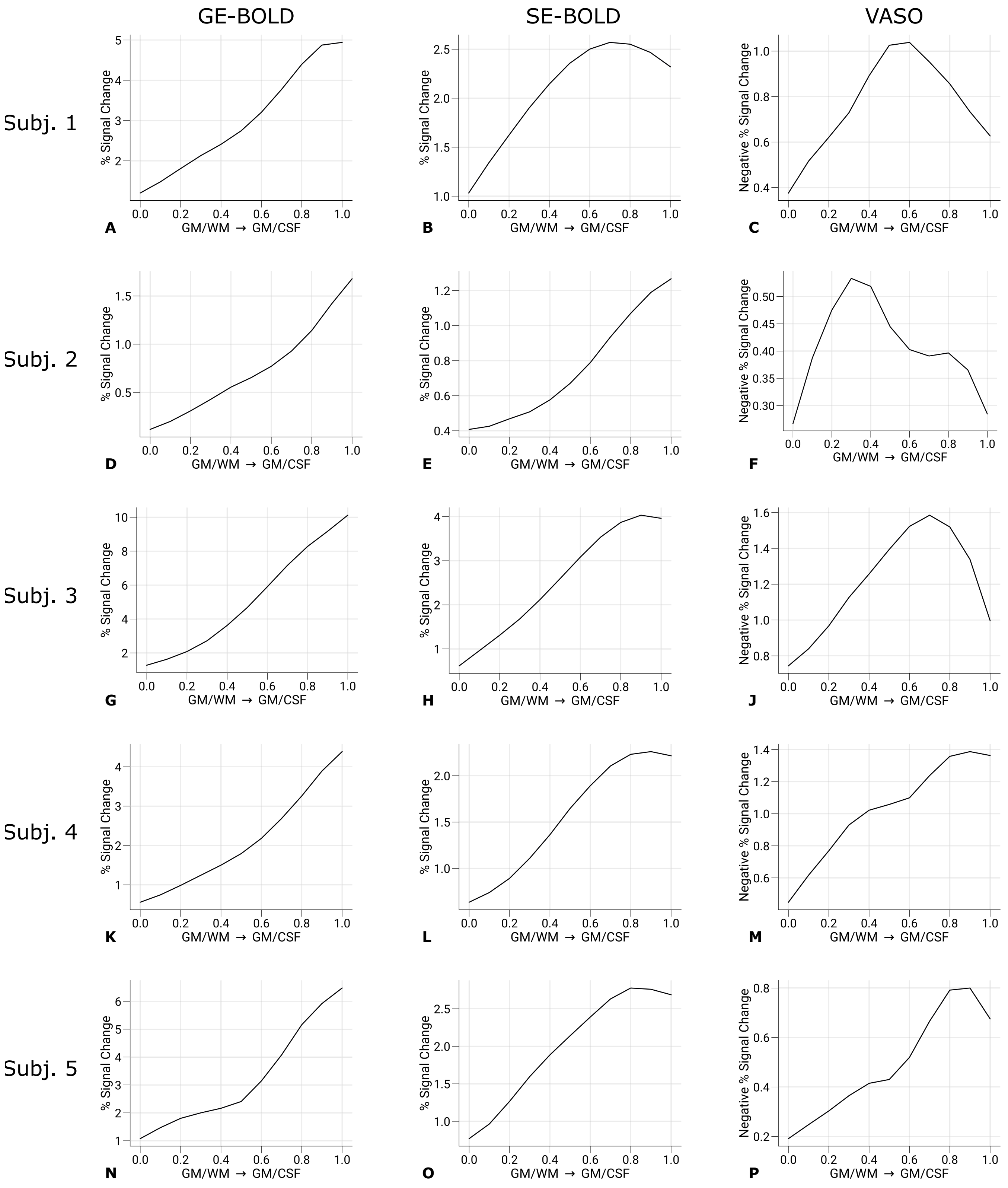

Supplementary Figure 8. Percent signal changes across cortical depth from single participants. Percent signal changes (contrast: left eye and right eye > baseline) for GE-BOLD (left column), SE-BOLD (middle column), and VASO (right column) are shown across cortical depth for single participants (average across two sessions). Only data points (n = 200) were used that were also selected for the decoding analysis. Note that we inverted the y-axis for VASO (right column) for easier interpretation. The variability of cortical profiles between participants can be identified.

bioRxiv preprint doi: https://doi.org/10.1101/2023.09.28.560016; this version posted August 27, 2025. The copyright holder for this preprint (which was not certified by peer review) is the author/funder, who has granted bioRxiv a license to display the preprint in perpetuity. It is made available under aCC-BY 4.0 International license.

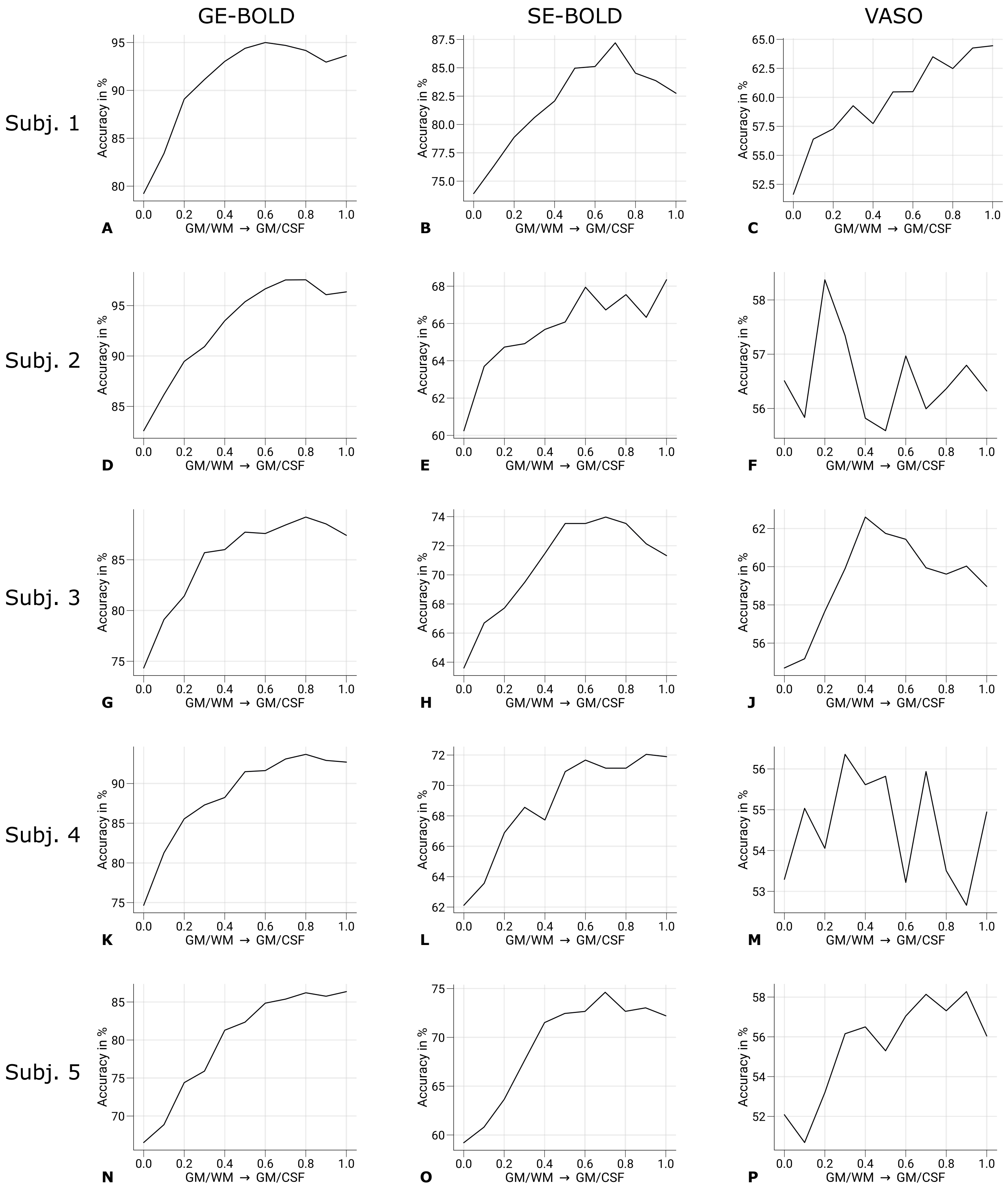

Supplementary Figure 9. Prediction accuracies across cortical depth from single participants. Prediction accuracies (prediction of the stimulated eye) for GE-BOLD (left column), SE-BOLD (middle column), and VASO (column) are shown across cortical depth for single participants (average across two sessions). The variability of cortical profiles between participants can be identified.

bioRxiv preprint doi: https://doi.org/10.1101/2023.09.28.560016; this version posted August 27, 2025. The copyright holder for this preprint (which was not certified by peer review) is the author/funder, who has granted bioRxiv a license to display the preprint in perpetuity. It is made available under aCC-BY 4.0 International license.

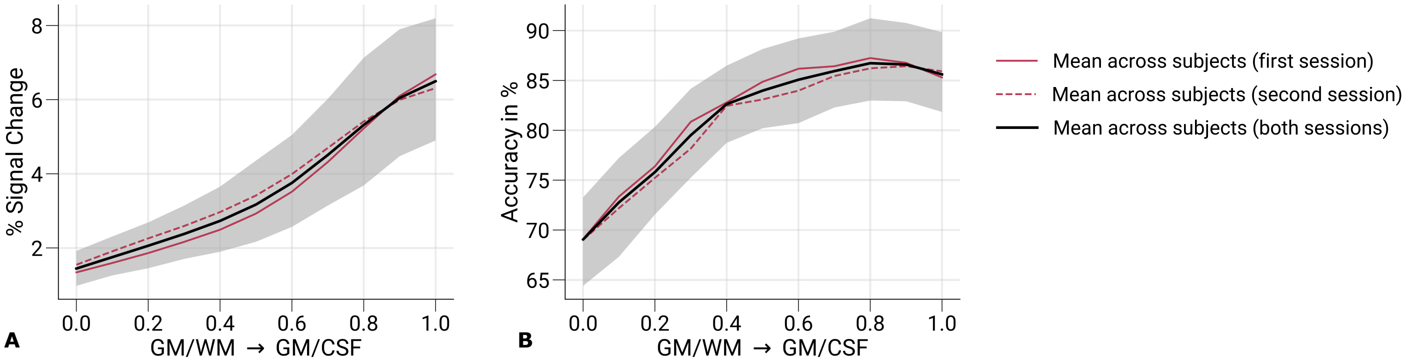

Supplementary Figure 10. Percent signal changes and prediction accuracies for not-nulled time points in VASO sessions. Mean percent signal changes (contrast: left eye and right eye > baseline) **(A)** and mean prediction accuracies (prediction of the stimulated eye) **(B)** are shown across cortical depth for not-nulled (BOLD-weighted) time series from VASO sessions. Red solid and dashed lines show the mean across participants from the first and second session, respectively. Black lines indicate the mean across participants and scanning sessions. The gray area demarcates the bootstrap 95% confidence interval (n = 1,000). Shapes of cortical profiles are similar to ***Figure 5A*** and ***Figure 6A***, respectively. Overall, lower prediction accuracies compared to ***Figure 6A*** might be attributable to the smaller temporal efficiency due to the longer TR in VASO acquisitions.

bioRxiv preprint doi: https://doi.org/10.1101/2023.09.28.560016; this version posted August 27, 2025. The copyright holder for this preprint (which was not certified by peer review) is the author/funder, who has granted bioRxiv a license to display the preprint in perpetuity. It is made available under aCC-BY 4.0 International license.

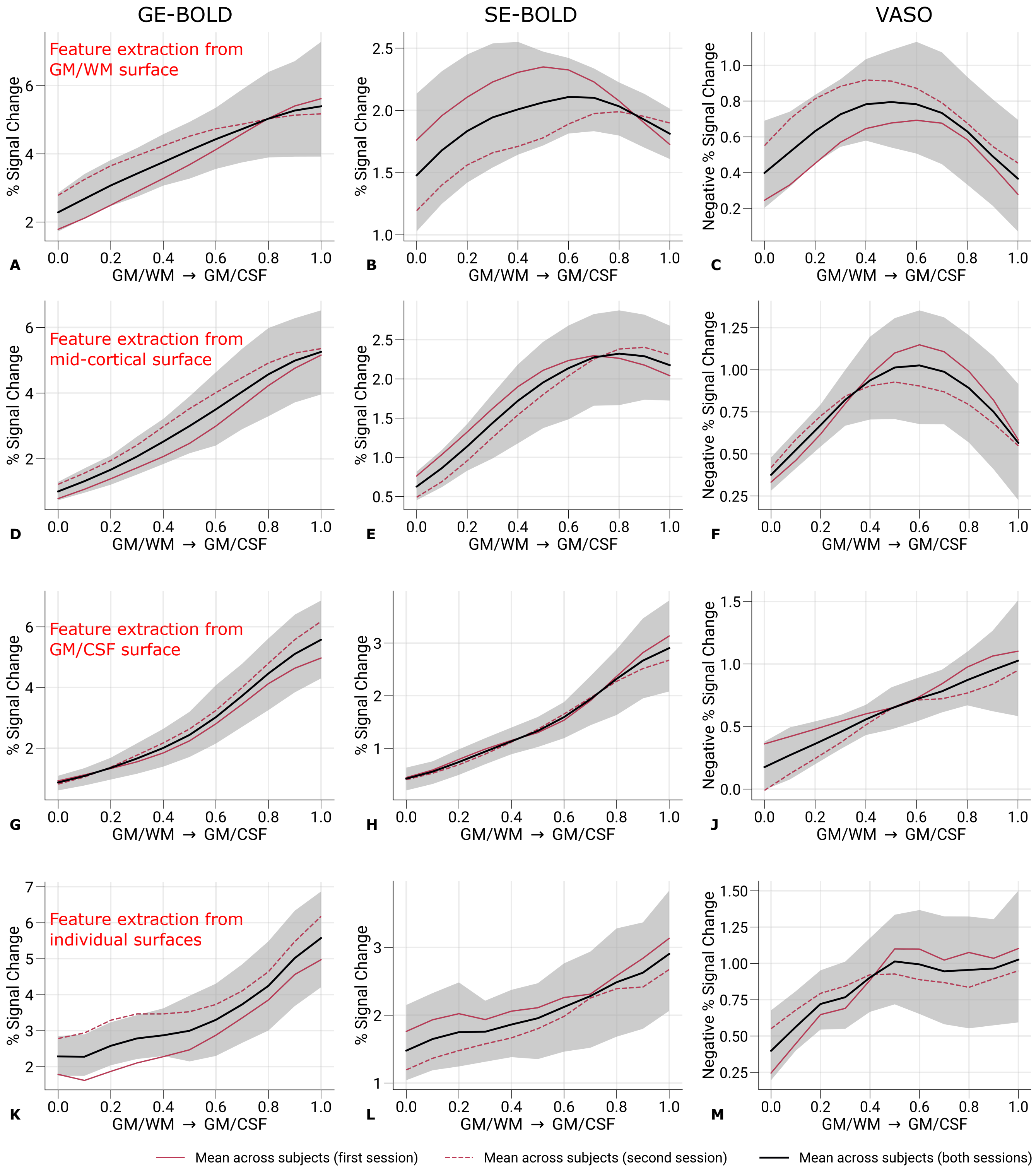

Supplementary Figure 11. Percent signal changes across cortical depth. Mean percent signal changes (contrast: left eye and right eye > baseline) for GE-BOLD (left column), SE-BOLD (middle column), and VASO (right column) are shown across cortical depth. In contrast to ***Figure 5***, features selection was restricted to data points sampled on the GM/WM **(A–C)**, the mid-cortical **(D–F)**, and the GM/CSF **(G–J)** boundary surfaces, respectively. In **K–M**, feature selection was performed for each cortical layer independently—other details as in ***Figure 5***.

bioRxiv preprint doi: https://doi.org/10.1101/2023.09.28.560016; this version posted August 27, 2025. The copyright holder for this preprint (which was not certified by peer review) is the author/funder, who has granted bioRxiv a license to display the preprint in perpetuity. It is made available under aCC-BY 4.0 International license.

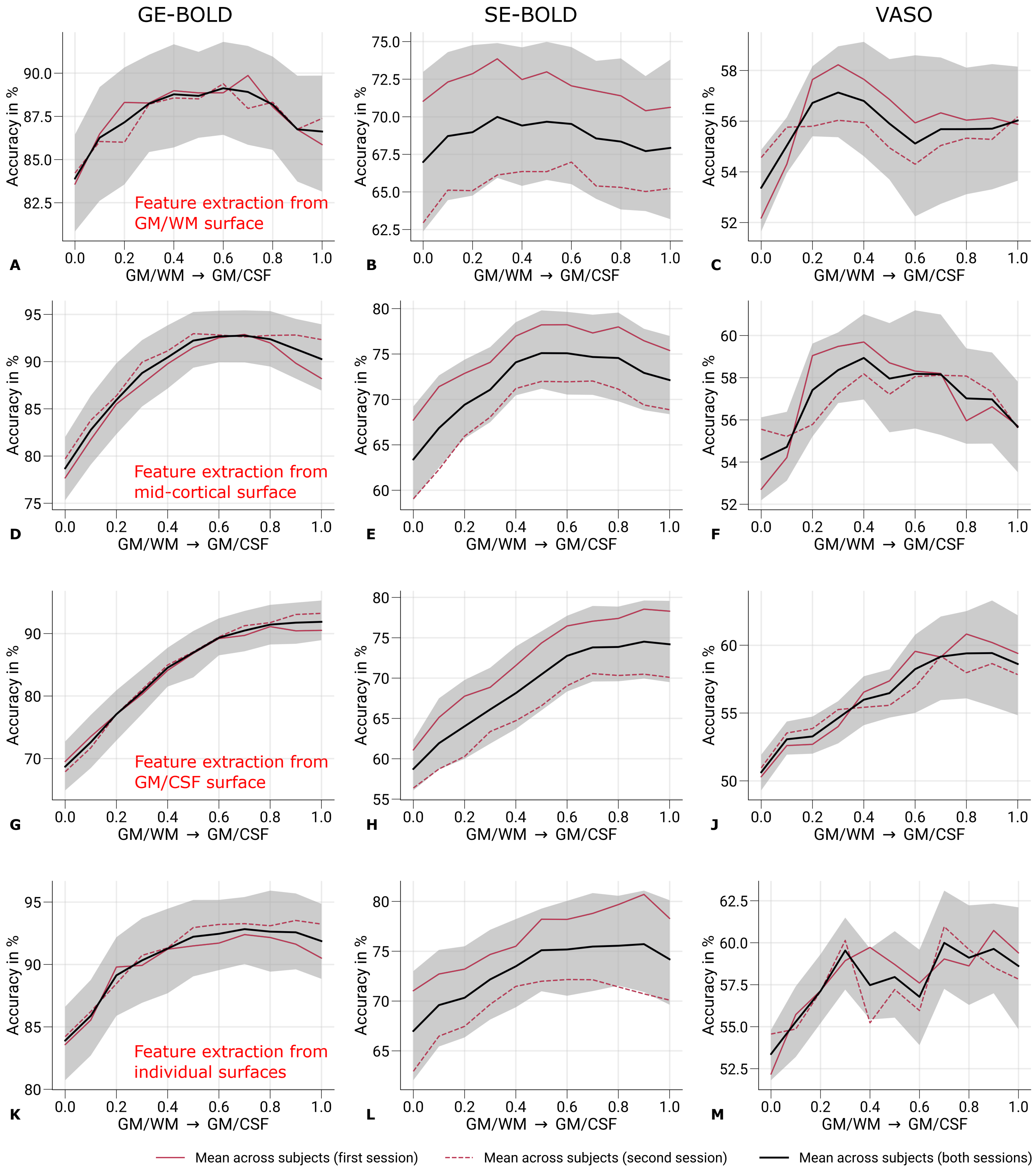

Supplementary Figure 12. Prediction accuracies across cortical depth. Mean prediction accuracies (prediction of the stimulated eye) for GE-BOLD (left column), SE-BOLD (middle column), and VASO (right column) are shown across cortical depth. In contrast to ***Figure 6***, features selection was restricted to data points sampled on the GM/WM **(A–C)**, the mid-cortical **(D–F)**, and the GM/CSF **(G–J)** boundary surfaces, respectively. In **K–M**, feature selection was performed for each cortical layer independently—other details as in ***Figure 6***.

bioRxiv preprint doi: https://doi.org/10.1101/2023.09.28.560016; this version posted August 27, 2025. The copyright holder for this preprint (which was not certified by peer review) is the author/funder, who has granted bioRxiv a license to display the preprint in perpetuity. It is made available under aCC-BY 4.0 International license.

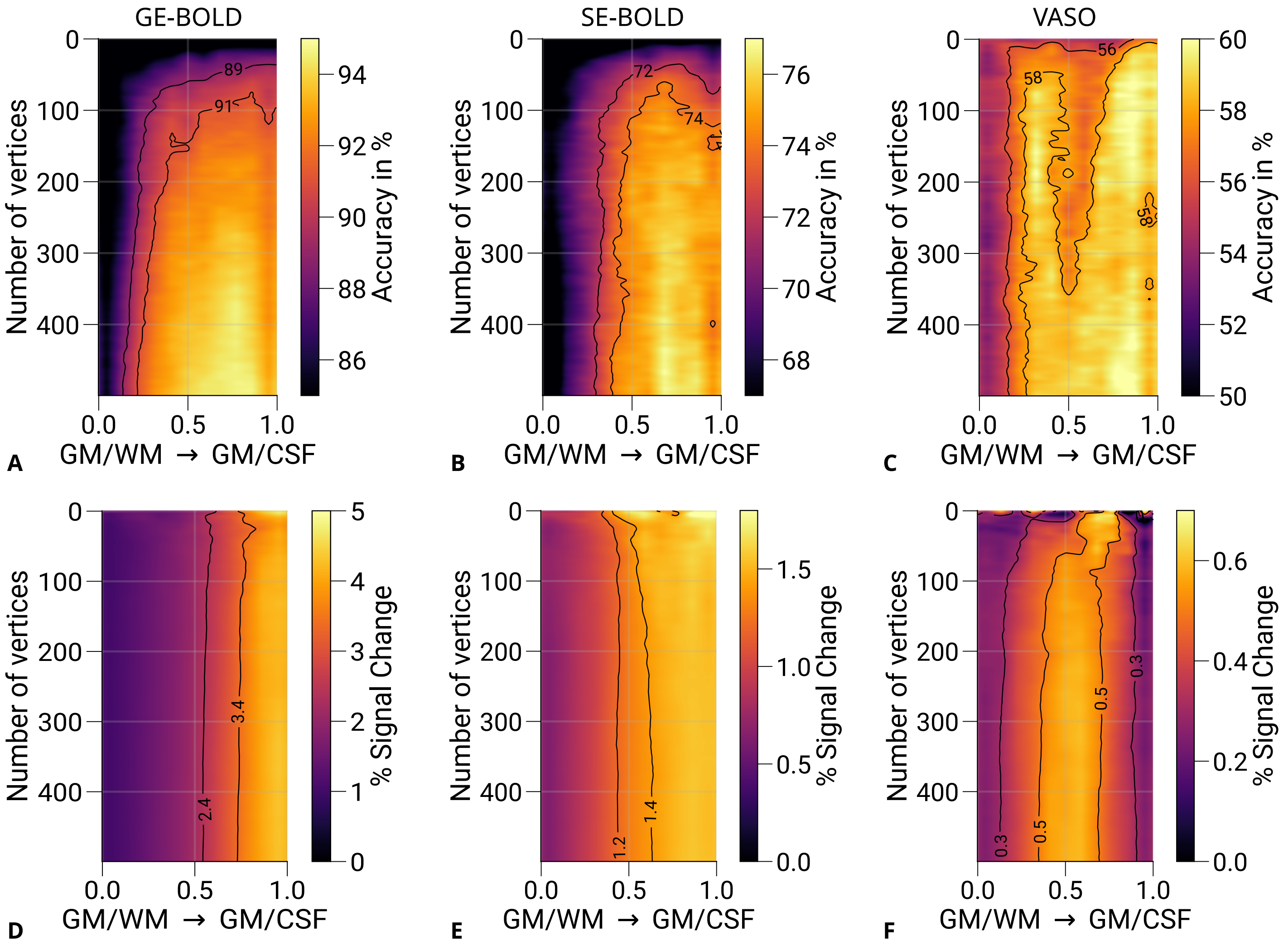

Supplementary Figure 13. Prediction accuracies and percent signal changes for different number of features. Mean prediction accuracies (prediction of the stimulated eye) for GE-BOLD **(A)**, SE-BOLD **(B)**, and VASO **(C)** are shown for a varying number of features (vertices) across cortical depth. **D–F** show corresponding percent signal changes (left eye and right eye > baseline) using the same data points. In contrast to ***Figure 7***, feature selection was performed for each cortical layer independently—other details as in ***Figure 7***.
